# Who should I involve in my research and why? Patients, carers or the public?

**DOI:** 10.1186/s40900-021-00282-1

**Published:** 2021-06-14

**Authors:** Kristina Staley, Jim Elliott, Derek Stewart, Roger Wilson

**Affiliations:** 1 TwoCan Associates, Ross on Wye, UK; 2 Independent Advocate for Patients in Health Research, Pembrokeshire, UK; 3 Patient Advocate, Nottingham, UK; 4 NCRI Consumer Forum, Sarcoma Patients Euronet, Shropshire, UK

**Keywords:** Patient public involvement

## Abstract

Patient and public involvement in research helps to make it more relevant and useful to the end-users. Involvement influences the design, delivery and dissemination of research, ultimately leading to better services, treatments and care. Researchers are therefore keen to involve patients, carers and public in their work, but are sometimes uncertain about who to involve. Some confusion may arise from the terms used. The UK’s catch-all term ‘patient and public involvement’ suggests this is a single activity, that perhaps both ‘patient’ and ‘public’ input are needed, or that either will do. The terms ‘patient’, ‘carer’ and ‘public’ have been defined, but are not used consistently. In fact there are many different contexts for involvement and many different kinds of decisions made, which then determine whose input will be most valuable.

Clarity about the ‘why’ can help answer the ‘who’ question. However, not all researchers are clear about the purpose of involvement. While it is often understood to have a moral purpose, or to improve research quality, this doesn’t always identify who needs to be involved. When learning is understood to be the purpose of involvement, then the most appropriate people to involve are those with relevant experiential knowledge. In research projects, these are people with lived experience of the topic being investigated. This could be patients, carers, members of the public or health professionals.

In this article we discuss how involving people who do not have the relevant experiential ‘lived’ knowledge may contribute to ineffective or tokenistic involvement. These people are as likely as researchers to make assumptions, risking missing key insights or resulting in outcomes that are off-putting or even harmful to research participants.

We conclude that greater attention needs to be given to the question of who to involve. Raising awareness of the significance of experiential knowledge and the contextual factors that determine whose input will be most useful will help everyone to understand their roles and improve the quality of involvement. It will help to maximise the opportunities for learning, increasing the likelihood of impact, and helping to achieve the ultimate goal of improved health and services.

## Plain English summary

Patient and public involvement in research helps to make it more relevant and useful to the end-users. Researchers are therefore keen to involve people but are sometimes uncertain about who to involve. Some confusion comes from the terms used. The UK’s term ‘patient and public involvement’ suggests there is only one activity and that both inputs are needed or either will do. The terms ‘patient’, ‘carer’ and ‘public’ are not used in the same way by everyone.

Involvement happens in many different situations, influencing different kinds of decisions, which then determines whose input will be most valuable. Being clear about the ‘why’ can help answer the ‘who’ question. However, not all researchers are clear about the purpose of involvement. When learning is understood to be the purpose, the most appropriate people to involve are those with relevant experiential knowledge. They provide insights based on their lived experience. In research projects, this is experience of the topic being studied. This could be patients, carers, public or health professionals.

We discuss how involving people who do not have relevant experiential knowledge may limit impact. These people may be as likely as researchers to make wrong assumptions. This risks missing key insights or making unhelpful decisions.

We conclude that greater attention should be given to the question of who to involve. Raising awareness of the importance of relevant experiential knowledge and other factors that determine whose input will be most useful, will help maximise opportunities for learning and increase the potential for impact.

## Background

Patient and public involvement in research helps to make research more relevant and more useful to the people who are directly affected by the results. Involvement influences the design, delivery and dissemination of research findings, which ultimately leads to better health and social care services, better treatments and better quality of care. Researchers and research institutions are therefore often keen to involve patients, carers and the public in their work, but are sometimes confused about precisely who to involve [1]. Sometimes they may choose the people they involve for pragmatic reasons, for ease and speed. However, not getting this right may contribute to ineffective or tokenistic involvement and may even cause harm [2]. In this article, we suggest that this issue requires much greater attention in order to improve the quality and impact of involvement and thus achieve the desired goal of improved health and care.

One of the challenges is that the terms ‘patient’, ‘carer’ and ‘public’ are not always used in the same way and the definitions that have been developed, may not necessarily aid understanding [3]. Researchers can still be left with the question ‘Which patients?’ or ‘Which public?’. Similarly, involvement is often described using the catch-all term ‘patient and public involvement’ suggesting there is only one type of activity and that perhaps both types of input are needed. This has also led to the creation of the acronym PPI and unhelpful terms such as ‘PPI representatives’. This further obscures the fact that there are very many different contexts for involvement, and these determine whose input will be most valuable. Simplifying the term to ‘public involvement’ may have added to the general confusion.

Perhaps a bigger issue is that the purpose of involvement is not always clear to all involved [4]. Having clarity about the ‘why’ is essential to answering the ‘who’ question. Involvement in research is sometimes understood to have a moral purpose [5], underpinning the view that people have a right to have their say in decisions that will have an impact on their lives. However, this does not explain who needs to be involved or precisely what they will do. Another commonly stated purpose is to improve the quality of research and to make it more relevant and useful to the end-users [5]. But this puts an emphasis on the end goal, without specifying who amongst the end-users can best help achieve it. It is therefore unsurprising that researchers remain unclear about who to involve in their work even when fully supportive of the rationale [1].

We suggest that there is not always a simple answer to the question ‘Who should I involve?’ as much depends on the context and the nature of the decisions being made. In this article, we draw on the involvement literature and our combined experience of promoting and supporting involvement to explore these different contexts and the implications for answering this fundamental question. Each of our 20+ years of experience includes active and continuing roles as involved patients/carers (JE/DS/RW), the development and management of patient involvement in research (KS/JE/DS/RW) including the early years of the National Institute of Health Research (NIHR) (RW/DS), and critical appraisal of involvement in a wide range of settings (KS/JE/DS).

This article mainly focuses on the issue of when patients, carers or public need to be involved in research projects. Similar questions are raised in relation to involvement in health and social care policy, but this is beyond the scope of this article. We note that the issue of who to involve raises many important and related questions such as what approaches to use to make involvement practical for different people, how to involve people who are seldom heard and how to make the process equitable so that none are excluded, but these larger topics merit articles in their own right and we do not discuss them here. Researchers will also have questions about how to find and recruit the most appropriate people, once they have been identified. This topic has been well covered in existing guidance [6] and therefore we do not address it in this article.

## Definitions of ‘patients’, ‘carers’ and the ‘public’

The following definitions are based on the work of Fredriksson and Tritter (2017) and McCoy et al. (2019) [7, 8]:

### Patient

Patients are people with health problems, who may be taking medicines or receiving treatment, or using health services. They are uniquely placed to provide insights in to what matters most to them and what could improve the quality of their lives. Their input helps to inform decisions about how best to meet their specific needs, whether that’s designing a health service or planning research. No one else has the relevant knowledge or experience to do this. Members of the public cannot be proxies for patients.

### Carer

Often a partner, family member, parent or friend, this person provides unpaid care for an individual with a health condition. Carers’ lives are thus affected by the health of the person they care for. They can provide a different perspective on what could improve the quality of life of people with a health condition, but may have distinct concerns and quality of life issues themselves. Carers should not be considered a proxy for patients (although this may be necessary in certain contexts) and are often involved in research in their own right.

### Public

The public includes ‘ordinary people in general, taxpayers or citizens’ without any particular interest or concern. The public’s role is to inform and influence decisions about how public money is spent, to ensure fairness, transparency and accountability. They are asked to consider the common good. They can help to solve complex social and ethical issues when multiple inputs are needed from diverse social groups. Although patients and carers are also citizens, they cannot be proxies for ‘the public’.

What these definitions lack is a reference to people’s experiential knowledge, the knowledge they gain through their lived experience. When learning is understood to be the purpose of involvement [9, 10], then the most appropriate people to involve are those with the most relevant experiential knowledge, which can sometimes be patients or carers or public or health professionals as we describe below.

## Who has the most relevant experiential knowledge?

The conversations between researchers and patients/carers/public that take place through involvement support a process of mutual learning [9, 10]. This helps researchers to avoid bias in their thinking resulting from their lack of experiential knowledge [11, 12]. Researchers would otherwise miss important information or make wrong assumptions. The people who do possess this knowledge can fill the gaps, identify potential problems and solve them, or, simply confirm that all is well, increasing researchers’ confidence and motivation.

Most often the people with relevant experiential knowledge are those with direct or indirect experience of the research topic being investigated or the area of healthcare under development. However, this includes very different kinds of people in different contexts [Table 1]. It is often patients and carers with an experience of a health condition, but sometimes it is members of the public who are only at risk of ill-health e.g. in public health research and service development [14]. Sometimes it is health or social care professionals, for example in research that aims to understand and improve their experience of service delivery [13].

**Table 1:** Examples of where people with different kinds of experiential knowledge are the most appropriate be involved in research projects

Topic of the research	People with relevant experiential knowledge to contribute through involvement
A clinical trial of a thumb splint for arthritis	People with arthritis of the thumbs (patients)
Testing the use of insulin pumps in pregnancy	Women with diabetes who have had children or wish to have children or have experience of using pumps during pregnancy (patients)
Evaluating new forms of psychological support for people affected by cancer	People with experience of different kinds of cancer (patients) Partners, relatives or friends of people who have had different kinds of cancer (carers)
Testing a new screening programme for lung cancer in people who smoke but have no symptoms	People who smoke but do not have lung cancer (a specific group of members of the public)
Exploration of the risk factors for hospitalisation with Covid-19	People from groups who have been amongst those who have been hospitalised more often with COVID-19 e.g. older people, people from BAME groups (specific groups of members of the public)
Testing new forms of support for distressed midwives	Midwives with experience of stress at work (health professionals) [13] Parents with experience of birth assisted by midwives under stress (a specific group of members of the public)
Reducing the risk of asthma in damp housing	Residents in a local community with experience of living in damp housing (a specific group of members of the public)
An intervention to increase up take of cervical screening by Asian women	Women eligible for cervical screening within the Asian community (a specific group of members of the public)
Testing a new skin cream for babies with eczema	Parents/ carers of babies with eczema (carers)
Evaluation of employment policies that aim to reduce health inequalities	Trade union representatives with experience of how their fellow workers fare under different policies [14] (a specific group of members of the public)

## Involving the ‘public’ in research

In our combined experience of decades of involvement, we have identified only one study where the research topic did not define a group with relevant experiential knowledge. This was a systematic review to assess whether people who have different blood pressure measurements in their right and left arms are at greater risk of heart disease or stroke, than people whose blood pressure is the same in both arms. There was no one who had any particular experience that would be relevant to this topic as it has implications for every adult who has their blood pressure measured by a health professional. The researchers therefore involved general members of the public in writing their funding application (personal communication).

We therefore conclude that when members of the public are involved in research, this almost always refers to the involvement of a specific group of people with a particular experience, rather than as ‘citizens’ lacking any specific interest at all. ‘Public’ in the context of involvement in research is therefore distinct in terms of purpose and roles, when compared to ‘public involvement’ in making public policy decisions. We believe problems arise if ‘involving the public’ is misunderstood to mean any member of the public can be usefully involved in research as described below. Current use of the term ‘public involvement’ may mean that the significance of involving people with relevant knowledge and experience is being lost.

## Does it matter if ‘members of the public’ are involved instead of ‘patients/carers’?

Marks et al. (2018) reported involving a member of the public in a research project about kidney disease, even though that person had no experience of the condition as either a patient or carer [15]. This involvement was reported to have many impacts including making the information sheets for potential participants easier to read and changing practical aspects of the research design. However, we question whether this approach is appropriate and the most effective form of involvement.

Staley et al. (2016) reviewed the comments made by a panel of people with mental health problems when reviewing participant information sheets for a wide range of research projects. While all members of the panel could comment on the language, tone and format of the information, there were some comments about the content which only people with relevant experiential knowledge could make [16]. For example, in a study of people with experience of psychosis that required participants to undergo an (Magnetic Resonance Imaging) MRI scan, it was only the person with experience of psychosis who was able to explain that if he was feeling paranoid, he would need to know exactly what piece of music would be played over the headphones while in the scanner, before he would agree to take part.

In another context (unpublished observations), when a member of the public with no experience of mental health problems commented on participant information for a mental health research project, they suggested removing the word ‘recovery’, because they believed ‘*no one ever recovers from a mental illness*’. This is a myth that the mental health community has been challenging for many years [17]. Such a change could have been off-putting or even offensive to the people with mental health problems who would be reading that information and may have prevented many from taking part.

On this basis, we conclude that members of the public have the same gaps in their knowledge and are just as likely to make the wrong assumptions as researchers. Involving members of the public who lack relevant experiential knowledge, risks missing important insights. Furthermore, it could result in outcomes that are inappropriate, off-putting or even harmful to research participants.

## Involving ‘patients and carers’ in research

Using the terms ‘patient’ and ‘carer’ to refer to the people with relevant experiential knowledge for involvement in research may also be unhelpful, because some people with this experience may not describe themselves as ‘patients’ e.g. mothers who have given birth, or smokers attending a lung-screening clinic. Similarly, friends, partners and family members may have considerable experience of how their lives and those of their loved ones are affected by a health condition, but not identify as a ‘carer’. These terms may be off-putting to the people who do need to be involved.

Nor is it a simple matter of involving any ‘patient’ or ‘carer’ with relevant experience of a health condition or of using services. Sometimes it may be important to involve people with a specific kind of experience of the health condition being researched, for example people who experience a particular kind of symptom. This is illustrated by the example reported by Staley (2016) at Parkinson’s’ UK [18]. The charity set up a demographically diverse group (different ages, ethnicities and gender) of people with experience of Parkinson’s disease to comment on research proposals. When this group commented on the design of a clinical trial of a medicine management device, they gave the researcher feedback that was very negative, the complete opposite of the views of the patients and carers she had previously consulted. The differences in opinion resulted from the fact that everyone in the Parkinson’s UK group had come to a meeting in London and were still able to travel, because their symptoms were mild. They wanted a small device they could carry around. The device was large and more suitable for people who were housebound. It was patients who were more severely affected who had previously expressed great enthusiasm for the device. Therefore, the Parkinson’s UK group, given the task of evaluating the research proposal, had not included people with the relevant experiential knowledge for this particular project. This could have caused the researcher and the charity to draw the wrong conclusions about the device’s potential.

Another complication is that researchers ‘don’t know what they don’t know’ [12] and therefore do not know at the outset which insights they need, or specifically who has them [10]. To maximise the opportunities for learning, it is important for researchers to ensure diversity of experience among the people they involve. This does not mean involving a representative sample of the affected population as is commonly misunderstood [19] and this has proved to be ineffective as described above. Having a diverse group based on their demographics may not always translate directly into a group with diverse experiences. Ensuring diversity amongst the people involved may mean considering factors in addition to or even instead of standard demographics to include people who are affected differently by the same illness [18]. The factors that need to be considered will depend on the research question and the nature of the decisions to be made. Importantly, people with experience of the topic can help to identify who needs to be involved and may challenge researchers’ assumptions about whose experience is most relevant.

## Contributions from patients, carers and the public beyond their experiential knowledge

When patients, carers or the public are involved in research, they may bring valuable skills and experience in addition to their lived experience. These skills come from other parts of people’s lives including their work, volunteering and family roles. However, it is the *combination* of experiential knowledge and other life skills that ensures effective involvement. For example, a person with multiple sclerosis (MS) who works as a conference organiser could also contribute planning skills to a research dissemination event *as well as* their experiential knowledge of MS. They could draw on their insights as a patient to design a programme that meets the practical needs of other patients (e.g. short sessions to address fatigue and cognitive problems), as well as identifying which aspects of the research findings are likely to be of most interest to the patient community. Someone who *only* had event planning skills and no experience of MS, would in effect be acting as a volunteer, a role that could be open to anyone.

Similarly, the contributions that patients, carers and the public make during their involvement can go beyond the insights they provide from their lived experience. Crocker et al. (2017) describe how involved patients, carers and the public can contribute in other ways, bringing perspectives that are different to professionals, finding solutions to problems that are ‘outside the box’, asking the simple but challenging questions that no one else dares ask, and changing the dynamics in the room just by being present [20]. These kinds of contributions may not depend on having relevant experiential knowledge, which might be interpreted to mean that any member of the public could then usefully contribute. However, people *with* the relevant experiential knowledge can contribute in all these different ways *as well as* providing valuable insights to challenge researchers’ assumptions. Involving people with relevant experiential knowledge is therefore optimal as it maximises the potential for learning and impact from involvement.

## When do the ‘public as disinterested citizens’ need to be involved in research?

The term ‘involvement in research’ is used to describe involving people in a wide range of research-related decisions from setting national policy, to allocating research funding, through to deciding the details of a research proposal. For all the reasons described above, we argue that involving the people with relevant experiential knowledge is essential in individual research projects. However, when research decisions need to reflect the broader public interest (e.g. when allocating public research funds across diverse areas of health research), or need to be informed by the public’s views on the social or ethical acceptability of the research (e.g. the acceptable research uses of NHS health data) [8], then it is appropriate to involve the public as disinterested citizens i.e. as people who are not prejudiced and do not have a direct interest in the outcome. In this context, patients and carers along with other experts such as health professionals and researchers, could provide information for public policy decision-makers to consider, but should not be able to bias the outcomes [8]. There is a risk that patients or carers acting in their own interest could make decisions that conflict with the interests of wider society.

Confusion about who to involve arises when the nature of a research decision is not clearly defined. For example, McCoy et al. (2018) state that patients should not be involved in priority-setting in research, as they could bias such decisions towards their own interests [1]. However, priority setting refers to more than one type of decision. When ‘priority-setting’ refers to identifying priority *topics* for research on a specific condition (e.g. within James Lind Alliance Priority Setting Partnerships) then patients/carers are in the unique position of knowing what research would most benefit them and patients/carers (not public) should be involved. Similarly, when it refers to how a charity that focuses on one condition decides how to allocate its research funding, then involvement of patients and carers with that condition is again essential. However, when priority-setting refers to deciding how to prioritise amongst different projects competing for public research funding, then disinterested citizens would be best placed to be involved.

## What does this mean in practice

We notice a tendency for researchers to address the question ‘Who to involve?’ by instead asking ‘Who do I need to invite to join a Patient and Public Advisory group for my project?’ The starting assumption is that a group is needed and that it somehow needs to be ‘representative’ of the population and diverse in terms of demographics. The researchers may go to great lengths and expense to include people from across the United Kingdom (UK), different ages, genders, ethnic backgrounds, patients as well as carers. While this does go some way towards to addressing the important issue of diversity amongst the people involved, it may not be necessary or useful in every context, as we have seen in the Parkinson’s example above.

In another example, in a workshop with such a group of parents who had experienced stillbirth, one of the mothers commented that there were other people who really needed to be involved. The group had been recruited to help with a study to develop support services for bereaved parents and they recognised that they had all coped well with their experience as they were now in a position to be able to attend a workshop. Some of them knew people who were still not functioning years after a stillbirth and would not have come to a workshop as they ‘still needed to visit their baby’s grave every day’. Importantly, the group not only identified this important gap but also came up with solutions, including being an intermediary to bring in those people’s views into the research design.

While it may not be possible to be prescriptive, as each case may need to be different, we would recommend the following broad approach to researchers to answer the question ‘Who do I need to involve?:

1. Consider who has direct experience of the topic being investigated and therefore who has relevant experiential knowledge – is this very specific to one condition, a number of conditions, specific health inequalities or cultural communities? Does stage of illness or experience of particular symptoms matter? Does it relate to a setting, or geographical place or type of care/ treatment received? What range of people might have relevant experience, including patients and carers, healthcare staff and members of the public?
2. Have some initial exploratory conversations with relevant patients/carers/public to get input on your research and your ideas about how and who to involve. Test out your assumptions about who has relevant experience in these conversations. Ask them who else may need to be involved. This could be done informally on the phone, over coffee etc.
3. Consider and develop a range of approaches to involving people that meet the practical and support needs of the people you have identified as important to involve. This might mean setting up a group that meets formally. It might mean working with patients and carers to reach people in their community that you might otherwise find difficult to involve. It might mean you going out into the community for example visiting people at home, in a care home or attending community group meetings, so that you can have conversations with the most appropriate people.
4. Ensure the approaches you use are fair and do not exclude people who have relevant experiential knowledge to contribute, including people who are seldom heard.

## Conclusion

The confusion around whether to involve patients, carers or the public in research is unsurprising given the inconsistency and variation in the way these terms are defined and used and the many different kinds of research decision such involvement hopes to influence. As with all questions about involvement in research, the context is crucial. No simple rules apply to every situation, but we have highlighted the importance of involving people with relevant experiential knowledge whether they are a patient, carer or a person without a health condition, whenever the intention is to make a research decision that genuinely reflects *their* interests. This is particularly important for individual research projects. The term ‘people with lived experience’ can usefully describe all those who have relevant experiential knowledge to contribute in this context.

In recent conversations with members of the public who have been involved in research *without* having any relevant experience, we have noticed they find it difficult to hear that their involvement might not be the optimum approach. From their perspective, they see themselves as having made a big difference to research, for example in making information written for patients much easier to read. However, as in the case of researchers, members of the public ‘don’t know what they don’t know’ and may not be aware of what they are missing or assuming about patients’/carers’ interests and values. Involving the public as citizens without any particular experience or interest only becomes important when the outcome of that decision needs to be made for the benefit of society as a whole. We have discussed this issue in relation to health research, but similar arguments can be made for research in social care.

Changing the definitions and terms used may be helpful to reduce confusion, but is unlikely to be sufficient to bring clarity. Such terms are rarely used consistently in any case. We conclude that everyone with an interest in involvement in research needs a clearer understanding of the significance of experiential knowledge and greater awareness of the factors that need to be considered when deciding precisely who to involve in any given context. This will not only help patients, carers and members of the public understand what they are expected to contribute, but will also ensure greater sensitivity and transparency around recruitment, by helping to explain why certain people are being selected. For researchers who are interested in how to improve the quality of involvement, this understanding will maximise the opportunities for learning and thus the potential for impact.

## Data Availability

Not applicable.

## Abbreviations

MRI: Magnetic Resonance Imaging
MS: Multiple Sclerosis
UK: United Kingdom
